# Gamma-tocotrienol, a radiation countermeasure, reverses proteomic changes in serum following total-body gamma irradiation in mice

**DOI:** 10.1038/s41598-022-07266-5

**Published:** 2022-03-01

**Authors:** Elliot Rosen, Oluseyi O. Fatanmi, Stephen Y. Wise, V. Ashutosh Rao, Vijay K. Singh

**Affiliations:** 1 Division of Biotechnology Research and Review III, Office of Biotechnology Products, Center for Drug Evaluation and Research, U.S. Food and Drug Administration, Silver Spring, MD USA; 2 Division of Radioprotectants, Department of Pharmacology and Molecular Therapeutics, F. Edward Hébert School of Medicine, Uniformed Services University of the Health Sciences, 4301 Jones Bridge Road, Bethesda, MD 20814 USA; 3 Armed Forces Radiobiology Research Institute, Uniformed Services University of the Health Sciences, Bethesda, MD USA

**Keywords:** Diagnostic markers, Predictive markers

## Abstract

Radiological incidents or terrorist attacks would likely expose civilians and military personnel to high doses of ionizing radiation, leading to the development of acute radiation syndrome. We examined the effectiveness of prophylactic administration of a developmental radiation countermeasure, γ-tocotrienol (GT3), in a total-body irradiation (TBI) mouse model. CD2F1 mice received GT3 24 h prior to 11 Gy cobalt-60 gamma-irradiation. This dose of radiation induces severe hematopoietic acute radiation syndrome and moderate gastrointestinal injury. GT3 provided 100% protection, while the vehicle control group had 100% mortality. Two-dimensional differential in-gel electrophoresis was followed by mass spectrometry and Ingenuity Pathway Analysis (IPA). Analysis revealed a change in expression of 18 proteins in response to TBI, and these changes were reversed with prophylactic treatment of GT3. IPA revealed a network of associated proteins involved in cellular movement, immune cell trafficking, and inflammatory response. Of particular interest, significant expression changes in beta-2-glycoprotein 1, alpha-1-acid glycoprotein 1, alpha-2-macroglobulin, complement C3, mannose-binding protein C, and major urinary protein 6 were noted after TBI and reversed with GT3 treatment. This study reports the untargeted approach, the network, and specific serum proteins which could be translated as biomarkers of both radiation injury and protection by countermeasures.

## Introduction

Intended or unintended exposure to high dose radiation exposure can result in devastating health consequences^1^. Hence, radiation medical countermeasures are an urgent and unmet need^2^. Acute radiation syndrome (ARS) occurs in humans following total-body or partial-body exposures to ionizing radiation with doses above 1 Gy at a high dose rate. ARS includes the hematopoietic (H-ARS; 2–6 Gy), gastrointestinal (G-ARS; 6–8 Gy), and the neurovascular (> 8 Gy) sub-syndromes (radiation doses are for humans)^3^. The neurovascular sub-syndrome is considered untreatable even with full supportive care and countermeasures^4^. Individuals exposed to radiation doses resulting in H-ARS and GI-ARS can respond to radiation countermeasures. Therefore, radiation countermeasures are being developed for these two sub-syndromes of ARS^5^.

Over the last 6 decades, efforts have been made to develop radiation medical countermeasures for ARS. However, in the U.S., only four agents are approved by the Food and Drug Administration (FDA) for H-ARS^6–16^. All four agents are radiomitigators for use *after* radiation exposure. To date, there is no approved radioprotector that can be used prior to radiation exposure for protection from the harmful effects of radiation exposure. Many natural products have been evaluated for the prevention and treatment of diseases and compared to their synthetic counterparts in preclinical and clinical studies^17,18^. Vitamin E is a family of antioxidant agents which regulate peroxidation reactions and scavenge free-radicals^19,20^. Vitamin E has eight different isoforms belonging to two subgroups: four saturated tocopherols (α, β, γ, and δ) and four unsaturated tocotrienols. These eight agents are collectively known as tocols. Several in vitro studies have demonstrated that tocotrienols are mechanistically stronger antioxidants than tocopherols^18,21–26^.

Gamma-tocotrienol (GT3) is an antioxidant and an inhibitor of 3-hydroxy-3-methylglutaryl-coenzyme A reductase, and a component of vitamin E^27,28^. It is under advanced development as a radioprotective countermeasure that can be used for pre-exposure prophylaxis^29^. GT3 has been demonstrated in several studies to increase survival against lethal doses of radiation inducing H-ARS in the murine model when administered prior to total-body irradiation (TBI), and its dose reduction factor has been estimated to be 1.29^30^. GT3 has also been shown to accelerate hematopoietic recovery in murine as well as nonhuman primate (NHP) models^30^. GT3 treatment has led to increased bone marrow hematopoietic progenitors in CD2F1 mice exposed to gamma-radiation^31^. Furthermore, GT3 induced high levels of some cytokines and growth factors, particularly granulocyte colony-stimulating factor (G-CSF). The administration of G-CSF antibody abolished its radioprotective efficacy^32^. In a previously published study, GT3 and another tocol, α-tocopherol succinate, were found to reverse jejunum protein expression changes in an H-ARS model^33^. Furthermore, GT3 has been shown to have H-ARS radioprotective activity in NHPs when administered 24 h prior to 5.8 Gy and 6.5 Gy TBI^34^.

Biomarkers may be used to measure the absorbed radiation dose and the drug dose conversion from animal models to humans. Thus, it is critical to identify, validate, and quantify biomarkers^35,36^. Several countermeasures are under advanced development following the US FDA Animal Rule^36,37^. Hence, it is vital to identify biomarkers for countermeasure efficacy that can be helpful to convert drug doses from animal model studies to those that are applicable when used in clinical settings^37–39^.

The differential protein expression in serum samples collected from mice treated with GT3 and exposed to total-body γ-radiation were analyzed. The serum samples were used for two-dimensional differential in-gel electrophoresis (2D-DIGE) coupled with mass spectrometry and advanced bioinformatics tools. Our objective for this study was to understand the specific biological pathways and networks regulating the radiation response and the molecular mechanism(s) underlying GT3 radioprotection using a total-body radiation dose of 11 Gy, which induces severe H-ARS and moderate GI injury in CD2F1 mice. The GT3 dose and treatment schedule in the proteomic study were the same, which provided 100% survival against 11 Gy TBI. We observed changes in the expression of 18 proteins in response to irradiation, and these changes were reversed as a result of prophylaxis with GT3. Such proteins could be translated as biomarkers for radiation injury and GT3 efficacy and warrant further studies.

## Results

**Figure 1:**
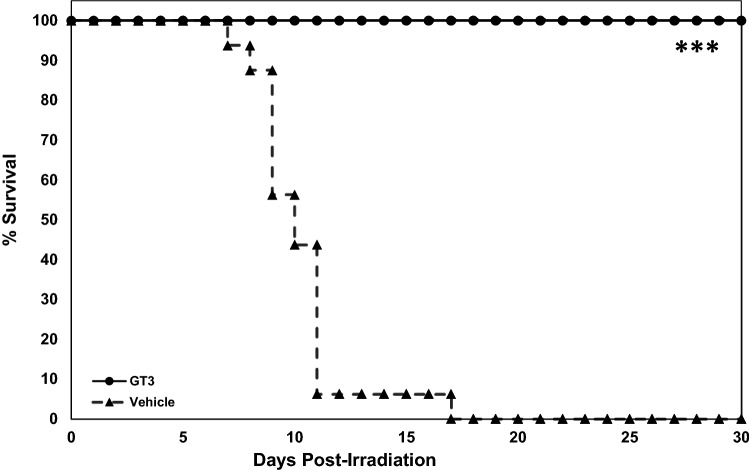
Efficacy of single GT3 (200 mg/kg) administrations on mouse survival after exposure to 11 Gy cobalt-60 gamma-radiation (0.6 Gy/min). CD2F1 mice (n = 16 per treatment group) were administered GT3 24 h prior to irradiation. Mice were observed for survival for 30 days post-irradiation. * denotes statistical significance between the GT3- and vehicle-treated groups, as determined by log rank test (***p < .001).

The purpose of this study was to report the comprehensive changes in mouse serum protein expression by GT3, a promising radiation countermeasure, and correlate these with survival against 11 Gy irradiation, which induces severe H-ARS and moderate GI injury. One group of mice (n = 16) was administered GT3 and the other group received vehicle 24 h prior to 11 Gy TBI, and all were observed for 30 days post-irradiation. Vehicle-treated animals started dying on day 7 and by day 17 post-irradiation, all animals in this group expired. On the other hand, all GT3-treated mice survived for 30 days until the experiment was terminated (Fig. 1, *p* < 0.001).

**Figure 2:**
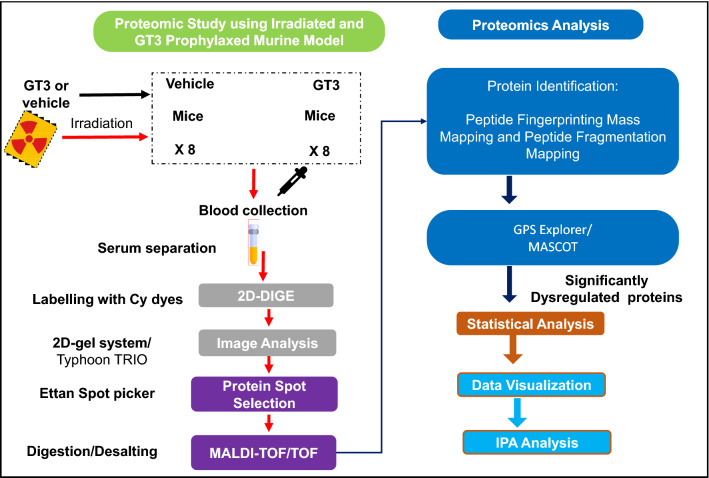
Study outline. Schematic of the animal study, sample preparation, and subsequent data analysis.

**Figure 3:**
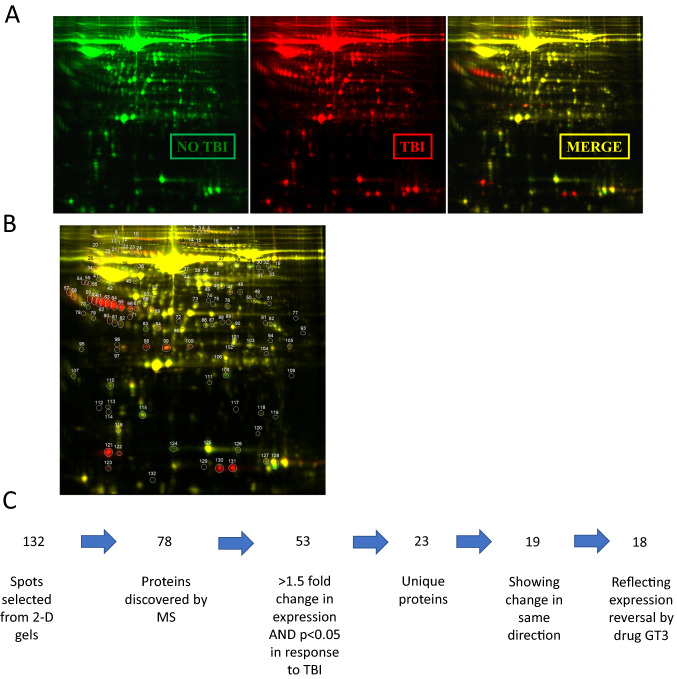
Detection and selection process for identifying proteins for proteomic analysis. (**A**) Lysine groups of serum proteins from three experimental samples were directly labeled with cyanine CyDye DIGE Fluor and run on same gel for 2D analysis. (**B**) Individual protein spots detected by selection software. (**C**) Proteins identified by MS were screened for change in expression, statistical significance, consistency in expression change, and uniqueness.

The molecular mechanism of this radioprotective agent was also investigated by considering the pathways and networks involved. The mice were prophylactically treated with GT3 or vehicle 24 h prior to supralethal TBI of 11 Gy. This TBI dose was selected because it is known to lead to severe H-ARS and moderate GI injury in CD2F1 mice. After 24 h post-irradiation, serum samples were collected. The 2D-DIGE method was employed to investigate changes in protein expression. Spot detection software was used to select 100–200 spots with isoelectric points ranging between pI 4 and 9, and molecular weights ranging from 10 to 150 kDa for trypsin digestion from the approximately 2500 proteins of interest from each gel (Fig. 2). The analyses identified proteins using MALDI-TOF/TOF and the MASCOT search engine (Fig. 3).

The gel analysis software identified 132 unique spots, of which 78 protein identities were determined. Of this cohort, 53 spots of these proteins exhibited a 1.5-fold or greater change in expression when comparing the serum samples of unirradiated animals to those from the TBI groups. The 53 spots represented 23 unique proteins because there were multiple instances of the same protein, likely the result of potential post-translational modifications. From these 23 proteins, 19 of the appearances reflected either a consistent increase or decrease in expression in response to TBI with respect to the unirradiated control group. Finally, 18 of these 19 proteins reflected an expression reversal when compared to the prophylactic GT3-treated group.

**Figure 4:**
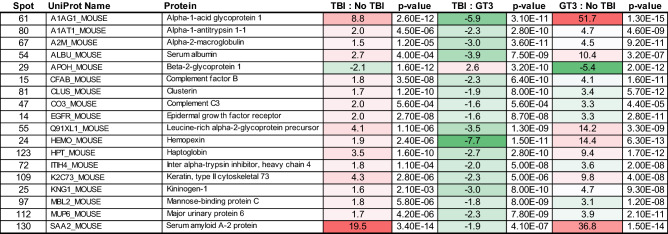
Serum proteins reflecting a change in expression in response to TBI and reversal with prophylactic treatment of GT3. Protein expression ratio changes are presented with a color scale to indicate increase (red) or decrease (green).

There were 18 proteins detected in the serum samples of the mice that received TBI and prophylactic treatment with GT3 that showed a marked change in expression which was reversed compared to non-prophylactic treatment with GT3, indicating a potentially protective effect of GT3 treatment (Fig. 4). Protein expression ratio changes are presented with a color scale to indicate increase (red) or decrease (green). One protein, beta-2-glycoprotein 1, showed a decrease in expression while the remaining 17 proteins exhibited an increase in expression in response to TBI with respect to the unirradiated group. With GT3 prophylactic treatment, beta-2-glycoprotein 1 showed an increase in expression while the remaining 17 proteins exhibited a decrease in expression, reversing the initial observation and indicating a treatment specific effect.

**Figure 5:**
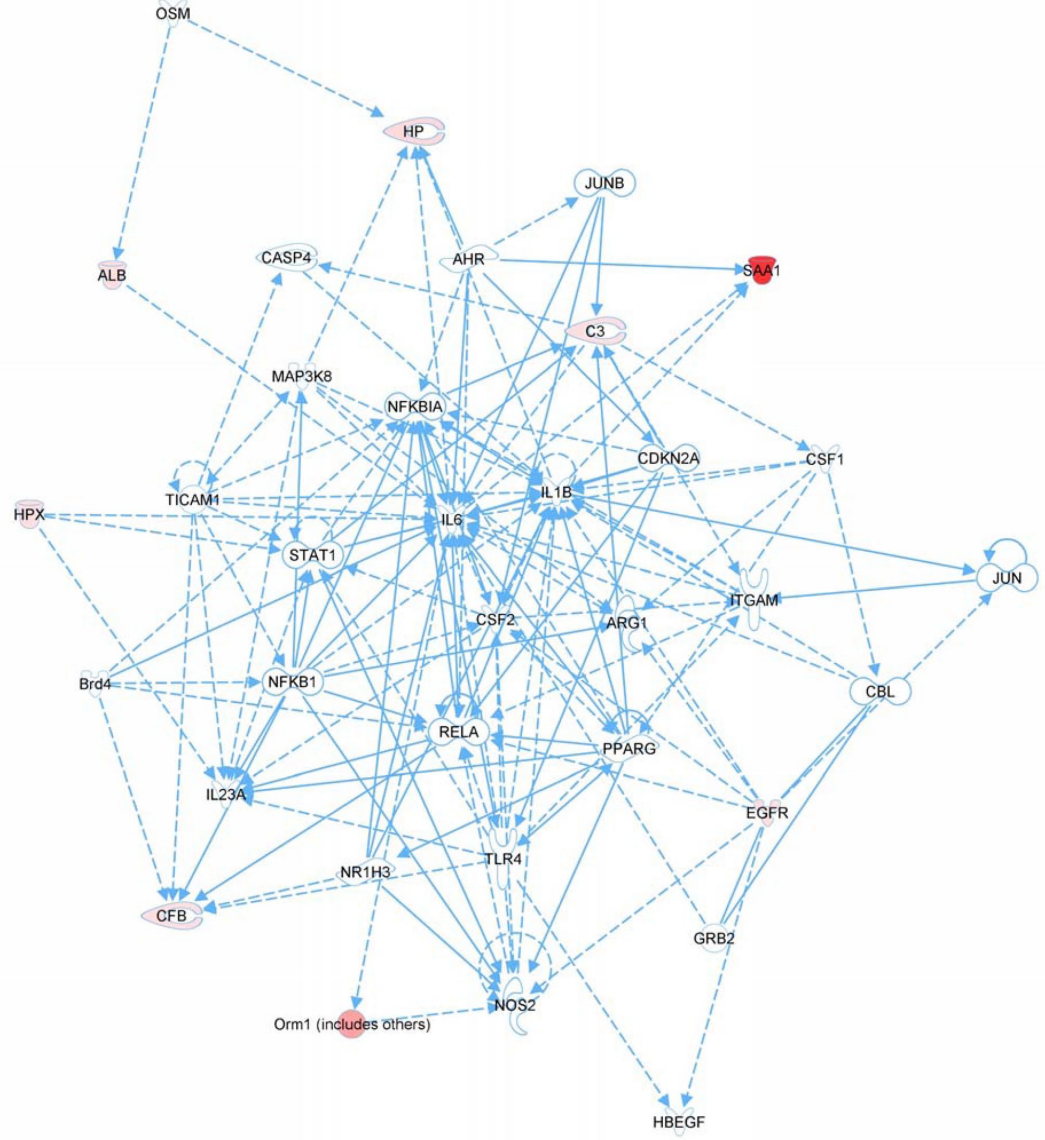
Network revealing relationships of serum proteins showing change in expression in response to TBI and reversal with prophylactic treatment of GT3. Ingenuity Pathway Analysis revealed proteins associated with cellular movement, immune cell trafficking, and inflammatory response. Proteins with red-fill backgrounds signify that these were down-regulated in response to TBI relative to no TBI. Proteins with no color-fill background were not identified in the MS analysis, but have a known relationship to proteins in the network.

The profile of serum proteins showed a change in expression in response to TBI with respect to the unirradiated group and then a corresponding reversal with GT3 prophylactic treatment were offered to the Ingenuity Pathway Analysis (IPA) software program to determine potential pathways and to chart a network based upon previously reported protein relationships (Fig. 5). The network suggested by IPA identified a number of potential relationships particularly associated with immune response involving interleukins, transcription factors, receptors, and apoptosis-related proteins.

## Discussion

GT3 has been found to be optimally effective in mice and NHPs when administered 24 h prior to irradiation^29,30,34,40^. A dose of 11 Gy total-body cobalt-60 gamma radiation exposure induces severe H-ARS and moderate GI injury. A GT3 dose of 200 mg/kg has consistently demonstrated radioprotective efficacy in the murine model^29^. We conducted GT3 survival as well as proteomics studies using the above treatment schedule at a dose of 200 mg/kg, radiation dose of 11 Gy, and CD2F1 mice. We observed 100% survival in the GT3-treated group by the end of the study, while all mice in the control group died by day 17 post-irradiation (Fig. 1). Suboptimal doses of GT3 (25 and 50 mg/kg) have also demonstrated moderate radioprotective efficacy in irradiated mice^41^.

Proteomic analysis has proven to be an essential tool for dissecting the mechanisms contributing to survival following exposure to ionizing radiation^42^. While tocols have shown the potential for radioprotection against ARS in studies, the underlying mechanisms and pathways of protection are not well understood^30,40^. The present study was designed to probe the serum proteins circulating following TBI and contribute new evidence of the specific proteins and networks involved in the radioprotection of GT3 treatment. We collected blood samples from animals at 24 h post-irradiation for proteomic analysis based on earlier studies where changes had been observed in serum cytokines and jejunum proteins at this time point^29,32–34^.

A total of 18 proteins found in the serum were determined to show a change in expression following TBI which was reversed with GT3 prophylactic treatment. Only one protein, beta-2-glycoprotein 1, showed a decrease in expression with TBI compared to the unirradiated group. Beta-2-glycoprotein 1, also known as ApoH, is a phospholipid binding protein which has anti-oxidant properties^43^. While the present study found beta-2-glycoprotein 1 declined with 11 Gy exposure, interestingly, Rithidech et al.^44^ found an increase in expression with a sub-lethal exposure of 3 Gy at 48 h. Of course, it is not surprising that the proteomic profile is temporally dynamic and dose dependent, making the case for further investigation into time course and irradiation dose to enhance our understanding of the proteomic dynamics which can inform biomarker identification.

The remaining 17 proteins identified showed an increase in expression following 11 Gy radiation. Nine of these proteins were also found to be elevated in expression by Rithidech et al.^44^ at 48 h following 3 Gy irradiation including alpha-1-acid glycoprotein 1, alpha-2-macroglobulin, clusterin, complement C3, hemopexin, haptoglobin, kininogen-1, mannose-binding protein C, and major urinary protein 6. While we found alpha-1-antitrypsin 1–1 to be elevated at 24 h, Rithidech et al.^44^ found expression decreased after 1 week with respect to the unirradiated control. This investigation used C57BL/6 mice and a 9.0 Gy (0.76 Gy/min) dose of total-body cobalt-60 gamma irradiation, which was approximately LD_40/30_, while the radiation dose in our study was supralethal leading to 100% mortality in untreated mice by day 17 post-irradiation.

Fu et al.^45^ found serum amyloid A, interleukin (IL)-22, urokinase, resistin, and IL-6 to be good predictors of mortality in a mouse model. This study used CBA/CaJ mice and a total-body sublethal dose of 3 Gy (0.72/Gy) Cesium-137 gamma-radiation. This study did not distinguish between the specific forms of serum amyloid A, but our study also revealed changes in serum amyloid A-2. While our study did not uncover the other proteins identified by Fu et al., it is likely that low abundant interleukins would be less likely to be detected by the 2-D gel method. Another recent study observing long term exposure to low-dose radiation found interleukin IL-5, IL-12p40, P-selectin, and serum amyloid A1 to be good predictors of low-dose radiation exposure with biodosimetric potential^46^.

The proteins identified in our study can be grouped into binding proteins, inflammatory response proteins, coagulation proteins/factors, protease inhibitors, and proteins involved in metabolism and growth. The binding proteins include beta-2-glycoprotein 1, hemopexin, haptoglobin, mannose-binding protein C, and major urinary protein 6. It should be noted that these binding proteins also play a role in inflammatory response^44^.

The inflammatory response proteins identified include alpha-1-acid glycoprotein 1, alpha-1-antitrypsin 1–1, inter alpha-trypsin inhibitor, heavy chain 4, serum amyloid A-2 protein, and haptoglobin. Interestingly, both alpha-1-acid glycoprotein 1 and serum amyloid A-2 protein have recently been reported as potential biomarkers of radiation injury, and these protein expression levels were reversed in our study with prophylactic GT3 treatment^47^. Seven of the proteins identified in our study can be classified as coagulation-related proteins. These members include alpha-2-macroglobulin, serum albumin, beta-2-glycoprotein 1, clusterin, hemopexin, haptoglobin, and kininogen-1. Of particular note is alpha-2-macroglobulin, which was recently reported as a potential radioprotectant in the esophagus in patients receiving thoracic radiotherapy^48^. Further evidence of radioprotection was previously reported in a rat model where the animals received 6.7 Gy X-ray TBI^49–51^. In these three studies, rats were exposed to total-body X-rays at a dose of 6.7 Gy (LD_50/30_), while the radiation dose used for our study (11 Gy) was supralethal.

Another group of proteins, the protease inhibitors, appeared to actively respond to irradiation injury, which was reversed with GT3 treatment. This group includes alpha-1-acid glycoprotein 1, alpha-1-antitrypsin 1–1, alpha-2-macroglobulin, and kininogen-1. It is important to note that we have demonstrated earlier that GT3 also restores gamma-irradiation-induced perturbations of miRNA (miR-30a-5p, miR-126-5p and miR-375) as well as metabolites (carnitine, propionylcarnitine, xanthine, and creatine) in a rhesus macaque NHP model^52,53^.

Other proteins detected fall under the metabolism and growth category. These include epidermal growth factor receptor, leucine-rich alpha-2-glycoprotein precursor, and keratin, type II cytoskeletal 73. Finally, we can classify two of the proteins identified as members of the complement system. These two proteins include complement factor B and complement C3.

It should be noted that there are study design limitations of this proteomic analysis. While the 2-D DIGE/MS approach resolves the multitude of data points, the limited throughput does not easily allow for evaluating multiple variables. Our results indicate further studies of variable irradiation dose, GT3 dose, and time points are warranted. Moreover, additional biological replicates can enhance the statistical significance of these findings. Additionally, we decided not to remove abundant serum proteins for this study, but this added step may be helpful in resolving proteomic changes of less prominent proteins.

Many of the proteins detected in the present study are known to be glycosylated. These proteins include alpha-1-acid glycoprotein 1, alpha-1-antitrypsin 1–1, alpha-2-macroglobulin, beta-2-glycoprotein 1, kininogen-1, hemopexin, and haptoglobin. While this study does not report on the glycosylation status of the serum proteins identified, this may be an interesting area of further inquiry. Changes in sialylation of glycoproteins following TBI and serum N-glycome following local irradiation exposure in mouse serum have been reported^54,55^. Site-specific glycosylation of seven major plasma proteins in response to partial-body irradiation therapy in cancer patients was recently reported^56^. This group included beta-2-glycoprotein 1, a protein found in the present study to show a reversible change in expression with GT3 treatment. However, the extent of the expression and time course exhibited variability between subjects. Clerc and collaborators have highlighted how N-glycosylation of human plasma proteins can significantly alter protein structure and function^57^. Furthermore, these changes can be considered consequences of disease state and can potentially serve as more precise biomarkers of disease. Five of the proteins identified in the current study found to be increased in expression with radiation exposure are major plasma glycoproteins including alpha-1-acid glycoprotein 1, alpha-1-antitrypsin 1–1, alpha-2-macroglobulin, beta-2-glycoprotein 1, kininogen-1, hemopexin, and haptoglobin. Therefore, we postulate that post-translational modifications, such as glycosylation may have utility in identifying more precise biomarkers of radiation exposure.

## Material and methods

### Animals and animal care

We procured 6 to 8-week-old male CD2F1 mice from Harlan Laboratories, Inc. (Indianapolis, IN, USA). Mice were kept in quarantine for 1 week, and microbiological examination confirmed the absence of *Pseudomonas aeruginosa*^33^. Mice were housed in an Association for Assessment and Accreditation of Laboratory Animal Care International accredited facility, eight per cage, in rooms maintained at 21 ± 2 °C, relative humidity of 50 ± 10%, with a 12 h light/dark cycle, and 10–15 cycles of fresh air per hour. Certified rodent rations (Teklad Rodent Diet, Harlan Laboratories, Inc.) and acidified water (HCl, pH = 2.5–2.8) were provided to the mice ad libitum. The study was performed according to the *Guide for the Care and Use of Laboratory Animals* of the Institute of Laboratory Animal Resources, National Research Council, U.S. National Academy of Sciences^58^. All animal procedures were completed according to a protocol (P-2011-04-002) approved by the Institutional Animal Care and Use Committee of the Armed Forces Radiobiology Research Institute/Uniformed Services University of the Health Sciences. This study is reported in accordance with ARRIVE guidelines.

### Experimental design

There were three groups: untreated and sham irradiated, vehicle-treated and irradiated, and GT3-treated and irradiated. There were eight mice in each treatment group for serum sample collection and proteomic analysis. In addition, there were two groups with 16 mice each for the survival study continuing for 30 d post-irradiation. The drug was administered 24 h prior to 11 Gy irradiation, which induces severe H-ARS in CD2F1 mice. This agent has been found to be optimally effective when administered 24 h before radiation exposure.

### Irradiation of mice

Irradiation boxes consisted of compartmentalized Plexiglas boxes designed to hold eight mice. Mice were exposed to a bilateral, midline dose of 11 Gy ^60^Co γ-radiation at a dose rate of 0.6 Gy/min, as described earlier^59^. Radiation dosimetry was established primarily on the alanine/EPR (electron paramagnetic resonance) system, currently accepted as one of the most accurate methods for relatively high radiation doses and was used for intercomparisons between national metrology institutions. The calibration curves (spectrometer e-Scan, Bruker Biospin, Inc., Madison, WI, USA) used in dose measurements are based on standard alanine calibration sets procured from the U.S. National Institute of Standards and Technology (NIST, Gaithersburg, MD, USA)^60,61^. The alanine dosimeters obtained from NIST had been calibrated in terms of absorbed dose to water using the US national standard radiation sources. Identical alanine dosimeters were placed midline within mouse phantoms (Plexiglas 1″ diameter, 3″ length) and irradiated for predefined periods of time. Measurement of their EPR signals were taken using the calibration curve constructed with alanine dosimeters from NIST-provided dose rates to water in the core bodies of mice. A small correction was subsequently applied for the difference in mass energy absorption coefficients between water and soft tissue.

### Drug preparation and administration

Yasoo Health, Inc. (Johnson City, TN, USA) supplied GT3 formulation in 5% Tween-80 in saline. GT3 formulation was adjusted to a final concentration for injection of 200 mg/kg in 0.1 ml total volume. Equal volumes of olive oil were administered as the vehicle (olive oil in 5% Tween-80 in saline)^33^. A Luer-Lock syringe with a 23 G needle was used for all drug administrations. Subcutaneous injections were administered at the nape of the neck 24 h before irradiation.

### Blood collection and serum separation

Blood was collected from anesthetized (Isoflurane, Abbott Laboratories, Chicago, IL, USA) mice via the inferior vena cava with a 23-gauge needle. After collection, blood was transferred to CapiJect serum separator tubes (3T-MG; Terumo Medical Corp., Elkton, MD, USA), allowed to clot for 30 min, and centrifuged at 400*g* for 10 min. The serum was collected and stored at − 70 °C until analysis as described earlier^37^. Based on earlier studies, the 24 h post-irradiation time point was selected for sample collection. At this time point, several cytokines and protein biomarkers have been reported to be induced in response to irradiation and GT3 prophylaxis in murine and NHP models^32–34^.

### Protein identification

To quantify changes in serum protein expression, Cy dyes (Cy3 or Cy5) were added to serum samples for labeling. Both eight vehicle control and GT3-treated serum samples were combined and labeled with Cy2, as previously described^33^. The 2D-DIGE separation method was employed in triplicate^62^. The Amersham Biosciences 2D-gel system (Amersham Biosciences, Piscataway, NJ, USA) was used to analyze the 2D-gels, and images were captured with the Typhoon TRIO and analyzed by ImageQuantTL software (GE Healthcare, Chicago, IL, USA). Decyder software version 6.5 (GE Healthcare) in combination with Ettan Spot picker (Amersham Biosciences) was used to select between 150 and 200 protein spots per gel for identification.

Digestion of the gel spots was performed in sequencing-grade modified trypsin protease at 37 °C before desalting and incorporation into the α-cyano-4-hydroxy-cinnamic acid matrix, which was then spotted onto a MALDI plate. The Applied Biosystems Proteomic Analyzer Spectrometer was used for matrix assisted laser desorption/ionization time of flight tandem mass spectrometry (MALDI-TOF/TOF) combined with peptide fingerprinting mass mapping, and peptide fragmentation mapping yielded specific protein identification. A GPS Explorer workstation equipped with MASCOT search engine (Matrix Science, Boston, MA, USA) was used to resolve peptide mass and associated fragmentation spectra from a primary sequence database.

Pathway and network analyses were prepared using IPA software (Qiagen, Redwood City, CA, USA) and were based on statistical significance as well as functional and biological relevance of the detected proteins. The IPA software was directed to focus on proteins that have the potential to appear in mouse serum for the purpose of more relevant pathway and network analyses.

### Statistical analysis

For survival data, a log-rank test was used to compare survival curves. Fisher’s exact test was used to compare survival rates at the end of 30 days. A Student’s *t* test was employed for all other statistical analyses and a threshold of 1.5-fold difference was set to determine a significant change in protein expression. Each data point is the result of three biological replicates with a *p* value of less than 0.05. A protein score confidence interval percentage or ion confidence interval percentage of greater than 95% was considered significant for the MALDI-TOF/TOF analysis.

## Data Availability

All data generated or analyzed during this study are included in this article.
